# Diversification rates, host plant shifts and an updated molecular phylogeny of Andean *Eois* moths (Lepidoptera: Geometridae)

**DOI:** 10.1371/journal.pone.0188430

**Published:** 2017-12-27

**Authors:** Patrick Strutzenberger, Gunnar Brehm, Brigitte Gottsberger, Florian Bodner, Carlo Lutz Seifert, Konrad Fiedler

**Affiliations:** 1 Department of Botany and Biodiversity Research, Division of Tropical Ecology and Animal Biodiversity, University of Vienna, Vienna, Austria; 2 Institut für Spezielle Zoologie und Evolutionsbiologie mit Phyletischem Museum, Friedrich-Schiller-Universität Jena, Jena, Germany; 3 Faculty of Science, University of South Bohemia, České Budějovice, Czech Republic; 4 Institute of Entomology, Department of Ecology, Biology Centre, The Czech Academy of Sciences, České Budějovice, Czech Republic; Institute of Plant Physiology and Ecology Shanghai Institutes for Biological Sciences, CHINA

## Abstract

*Eois* is one of the best-investigated genera of tropical moths. Its close association with *Piper* plants has inspired numerous studies on life histories, phylogeny and evolutionary biology. This study provides an updated view on phylogeny, host plant use and temporal patterns of speciation in *Eois*. Using sequence data (2776 bp) from one mitochondrial (COI) and one nuclear gene (Ef1-alpha) for 221 *Eois* species, we confirm and reinforce previous findings regarding temporal patterns of diversification. Deep diversification within Andean *Eois* took place in the Miocene followed by a sustained high rate of diversification until the Pleistocene when a pronounced slowdown of speciation is evident. In South America, *Eois* diversification is very likely to be primarily driven by the Andean uplift which occurred concurrently with the entire evolutionary history of *Eois*. A massively expanded dataset enabled an in-depth look into the phylogenetic signal contained in host plant usage. This revealed several independent shifts from *Piper* to other host plant genera and families. Seven shifts to *Peperomia*, the sister genus of *Piper* were detected, indicating that the shift to *Peperomia* was an easy one compared to the singular shifts to the Chloranthaceae, Siparunaceae and the Piperacean genus *Manekia*. The potential for close co-evolution of *Eois* with *Piper* host plants is therefore bound to be limited to smaller subsets within Neotropical *Eois* instead of a frequently proposed genus-wide co-evolutionary scenario. In regards to *Eois* systematics we confirm the monophyly of Neotropical *Eois* in relation to their Old World counterparts. A tentative biogeographical hypothesis is presented suggesting that *Eois* originated in tropical Asia and subsequently colonized the Neotropics and Africa. Within Neotropical *Eois* we were able to identify the existence of six clades not recognized in previous studies and confirm and reinforce the monophyly of all 9 previously delimited infrageneric clades.

## Introduction

Host plant affiliations have been recognized as a major driver of radiations among phytophagous insects ever since Ehrlich & Raven's seminal paper on co-evolution [1]. While instances of strict co-speciation and reciprocal co-evolution have rarely been found among folivorous insects, a growing number of case studies based on well resolved and dated phylogenies reveals that host plant shifts are important drivers of radiations [2,3], thereby contributing to the enormous species richness of megadiverse clades of phytophagous insects, for example among beetles [4] or butterflies and moths [5]. The recent advent of ever more powerful methods to estimate diversification rates from molecular phylogenies has set the stage for explicitly testing whether host plant shifts are indeed related to adaptive zones (e.g. [5,6,7]), and accompanied by elevated speciation rates [8]. However, the number of published studies on radiation dynamics in tropical, speciose clades of plant-feeding insects is still low.

*Eois* Hübner is one of the most species rich genera of geometrid moths in the subfamily Larentiinae. The genus currently comprises 254 validly described species, 211 of them (83%) occurring in the Neotropical region, 12% in the Oriental-Australian region, and 5% in Africa [9]. It is expected that only a small fraction of the true richness has been taxonomically described so far [9,10]. A previous molecular phylogeny of *Eois* by [11] provided strong support for the monophyly of the genus *Eois* as a whole, as well as for the clades formed by the Neotropical and Old World members of *Eois*, respectively. *Eois* moths have been the focus of rearing programmes and host plant studies [12,13,14,15]. They also were central to studies on tritrophic relationships [16], on interactions with host plant secondary metabolites [17,18], on general biodiversity patterns in the Andes [10,19,20], on biogeographic and taxonomic description patterns [9] and they served as a model for a case study on sequencing of old type specimens [21]. Therefore, re-visiting their phylogeny and evolutionary biology in the light of increased knowledge is of interest to a range of biological disciplines.

The tropical Andes are known to harbor extremely high species richness across many groups of plants [22], vertebrates (e.g. [23,24]) and arthropods (e.g. [25,26,27]). A complex interplay of drivers such as species interactions, and recent as well as long-term changes in climate and geological history, is likely responsible for Andean species richness and its uneven distribution in space. Previous molecular dating and analysis of diversification rates over time [28] indicated that *Eois* diversified in the second half of the Miocene and the Pliocene. More recent diversification during Pleistocene glacial cycles was found to be virtually absent. Periods of highest diversification rates within *Eois* were concurrent with both the earlier, central and the later, northern Andean uplift. Andean uplift took place in a south to north pattern with several distinct periods of accelerated uplift during the Neogene [29,30,31]. First the central Andes underwent a period of rapid uplift between ten and six million years ago which was followed by a more recent period of accelerated uplift in the northern Andes between five and two million years ago. Diversification rates in Neotropical *Eois* were found to follow a density dependent model with a pronounced slowdown towards the present. This same pattern was found in a number of studies on Neotropical moths and butterflies (e.g. [32,33]), while in temperate systems in the Nearctic and even more so in the Palearctic a major contribution of recent radiations to current species richness is evident [34].

We distinguish four major hypotheses explaining the origin of Neotropical species diversity:

1. Museum hypothesis: The museum hypothesis pertains to lineages whose species diversity is positively correlated with their age. This implies that there were no major changes of diversification and extinction rates during the lineage’s history [35,36]. The museum hypothesis is synonymous with the time-for-speciation hypothesis [37].
2. Relict hypothesis: Scenarios where lineages diversified early and subsequently experienced a net loss of species diversity due to increased extinction or decreased speciation rates are often lumped into the museum hypothesis e.g. [38]. We suggest that lineages subject to this scenario should rather be referred to as relicts in order to differentiate this very different pattern from the museum hypothesis sensu stricto.
3. Neogene diversification: This pattern applies to lineages that diversified during the Neogene followed by a slowdown of diversification in the Quaternary [35].
4. Quaternary diversification: Vicariant speciation in Pleistocene refugia has been proposed as the source of Neotropical diversity early on [39]. This scenario requires net diversification rates in the Quaternary to be higher than prior to the Quaternary [35].

Larvae of many species of Neotropical *Eois* are known to feed on *Piper* (Piperaceae) plants, in fact the moth genus is often being referred to as specialized feeders on *Piper* plants [17,40]. This frequently encountered trophic relationship of *Eois* with *Piper* has received considerable attention [12,14,17,40,41]. Close co-evolution of *Piper* plants, *Eois* moths and larval parasitoids has been postulated [14,17,28,41] but has never been conclusively demonstrated due to limited taxon sampling especially of *Piper* plants, see also [16]. First doubts on this exclusive trophic relationship with *Piper* were cast by [11] who collated isolated feeding records on other genera of Piperaceae and even isolated occurrences of caterpillars on other plant families. This skeptical view was subsequently corroborated by [15] who found ten *Eois* species in a relatively small study area in southern Ecuador feeding on the genus *Peperomia* (Piperaceae), while [42] reported the first records of *Eois* caterpillars on completely unrelated *Hedyosmum* (Chloranthaceae) plants from the same region.

Here we provide an updated perspective on the evolutionary history of *Eois* with a twice as dense taxon sampling compared to [13]. Even more importantly, the number of species with available host plant records has been increased sevenfold since the publication of our previous analysis. Specifically, we test (a) whether a denser taxon sampling reinforces previously identified temporal diversification patterns, and (b) whether this enlarged data set enables the detection of stronger phylogenetic signal with regard to the role of larval host plant affiliations on *Eois* radiations.

## Material and methods

### Collection of specimens and DNA sequencing

We used previously published sequences [13] and newly acquired mitochondrial COI and nuclear Ef1a sequences for an additional 109 Operational Taxonomic Units (OTUs). *Eois* moths used in this study were collected in 2005 within the Reserva Biológica San Francisco in southern Ecuador in an area of ca. 2.5 km^2^ (03°58.5’–3°59.7’S, 79°04.1’–79°05.1’W, 1800–2700 m a.s.l.). Additional sampling was carried out between 2008 and 2013 in adjacent areas of the Podocarpus National Park (1020–3021 m, see [20]). A small number of samples collected since 2002 at various localities in Costa Rica were also added to the data set.

Collection and export of specimens was done in accordance with the applicable laws and regulations. The Ministerio del Ambiente, Loja (Ecuador) granted the following research permits: 010-IC /DANVS/VS; 010-IC /DANVS/VS; No. 002-PNP-DBAP-RLZCH/MA; No. 002-2005-DBAPVS-RLZCH/MAE; 0014-IC-FAUNA-DRLZCH-VS-MAE; 016-IC-FAU-DPL-MA. Research in Costa Rica in the years 2003 and 2004 was performed under Resolucion No 125-2003-OFAU granted by the Ministerio de Amibiente y Energía.

Single legs of moths were homogenized with ceramic beads using a Precellys 24 homogenizer set to 5000 min^-1^ for 2x20 s. DNA extraction was performed with the DNEasy Tissue Kit (Qiagen), the Peqgold Tissue DNA mini Kit (Peqlab) according to the protocol supplied with the respective kit. Extraction of older specimens was performed by non-destructive extraction of DNA from abdomens [21]. The target fragments were amplified using the Fermentas PCR system. Target regions were the nuclear gene coding for translation elongation factor 1-alpha (Ef1α) and the mitochondrial gene coding for cytochrome oxidase subunit I (COI) (see S1 Table for primers). PCR reactions were set up with 2.5 μl of 10x (NH_4_)_2_SO_4_ PCR Buffer, 2 μl 25mM MgCl_2_, 0.1μl 10mM dNTPs, 0.5 μl of each primer (10μM), 1 μl dimethylsulphoxide (DMSO; only for Ef1α reactions), 1μl genomic DNA, 1 unit Taq Polymerase and filled to 25μl with PCR grade H_2_O. PCR reactions were purified by digestion with FastAP Thermosensitive alkaline phosphatase and Exonuclease I for 45 min at 37°C followed by 15 min at 80°C for enzyme deactivation. Sequencing reactions were set up with 1 μl ABI BigDye 3.1, 1μl primer, 1.5 μl of sequencing buffer, 1–2 μl template DNA and filled to 10 μl with PCR grade H_2_O and sequenced on an ABI capillary sequencer. All gene fragments were sequenced in both directions. PCR and sequencing primers as well as thermal cycler programs are indicated in S1 Table.

### Selection of taxa for phylogenetic analyses

Specimens for inclusion in this study were selected from a pool of approximately 800 *Eois* barcode sequences. We used the BIN system [43] as a provisional taxonomic hypothesis and selected one specimen per BIN in order to assemble our final dataset. We use the term ‘species’ for these taxonomic entities in the following. Outgroup sequences were taken from published data [11,44] We included 221 taxa of *Eois* plus 55 outgroup taxa in our dataset for a total of 276 taxa. Maximum likelihood trees were rooted with *Archiearis parthenias* which was recovered as the first branching taxon in the sister clade of Larentiinae+Sterrhinae by [44]. See S2 Table for a full list of included taxa and Genbank accession numbers of associated sequences.

### Sequence data processing

Proofreading of sequences and contig assembly was done with DNAStar Lasergene SeqMan Pro ver. 8. Heterozygous positions were coded as ambiguities. Assembled sequences had a length of 1220 bp and 1066 bp for COI and Ef1**α** respectively. All sequences were aligned using the all-in-one version of MAFFT v7.273 [45] on auto settings. Sequence data were prepared for analysis using the R package ‘ape’ [46]. Sequences were screened for unusual nucleotide composition and the presence of erratic stop codons to control for possible pseudogene amplification.

### Phylogenetic analysis

PARTITIONFINDER v1.1.1 [47] was used to determine the best-fitting partitioning scheme for our dataset. We performed a search of all possible partitioning schemes and all evolutionary models that can be implemented in BEAST resulting in 24 models of evolution being tested for 203 partitioning schemes. The best-fitting partitioning strategy was a six-partition scheme where all three codon positions of each gene were partitioned separately. The GTR+I+G model of evolution was applied to all partitions except the first codon position of Ef1a where a TrN93+I+G model was applied. Trees were inferred using BEAST 1.8.2. [48], Input files were generated using BEAUTi 1.8.2. Site models were unlinked among all partitions. Log-normal relaxed clock models were estimated with two partitions for COI and Ef1a respectively. Tree models were linked among all partitions. Site models were set according to the best-fitting model determined by PARTITIONFINDER with empirical base frequencies selected. In order to obtain time calibrated trees we used two calibration points. We calibrated the root of the tree with a normal prior (mean = 54.4, s = 5) based on the results of [49]. The age of the Larentiinae was constrained with a log-normal prior (mean = 2, s = 0.4232, offset = 34.06) based on the minimum age of the fossil *Geometridites larentiiformis* [50]. All other priors were left at default values with the following exceptions. COI.ucld.mean and Ef1a.ucld.mean were set to values known from previous analyses of subsets of the current dataset. We applied an exponential prior to both with a mean of 0.006 and 0.017 for COI and Ef1a respectively.

Chain length was set to 1.1x10E8 with states being sampled every 10000^th^ state. Computation was performed using the BEAGLE 2.1 library [48]. As recommended by [51] we ran BEAST while sampling from the prior only to rule out undesirable interaction among priors as well as ascertain that our time-calibration priors accurately represented the underlying data. Resulting log files were examined with Tracer v1.6 for convergence and satisfactory ESS values (>200). The first 10E7 states were removed as burn-in resulting in a sample of 10000 trees. TreeAnnotator 1.8.2. was used to determine the maximum clade credibility tree and median heights were annotated onto the tree. Maximum likelihood analyses were performed with RAxML 8.2.1 [52]. The dataset was partitioned according to the preferred strategy selected by PARTITIONFINDER. The GTR+G substitution model was applied to all partitions. A search for the best-known likelihood tree was performed with 100 replicates, automatic determination of rearrangement settings. Bootstrapping was done using the rapid bootstrapping algorithm implemented in RAxML with the number of replicates set to the autoMRE option and the remaining parameters and partitioning as stated above. Bootstrap support values were then drawn onto the maximum clade credibility tree obtained from BEAST. Trees were parsed and plotted using the R packages ‘ape’, ‘ggtree’, ‘ips’ and ‘OutbreakTools’.

### Analysis of diversification rates

BEAST tree samples were parsed with the R package ‘OutbreakTools’ and LTT plots were created with the ‘ltt.plot’ and ‘mltt.plot’ function of the package ‘ape’. We plotted the entire tree sample for Neotropical *Eois* as well as each recognized internal clade containing at least 10 species. The fit of several rate-constant (pure-birth, birth-death) and rate-variable models (DDL, DDX, yule2rate, yule3rate) was tested using the ‘fitdAICrc’ function in the R package ‘laser’ [53]. Input trees for ‘fitdAICrc’ were parsed using ‘read.beast’ in the package ‘ips’. Gamma statistics were calculated with ‘gamStat’.

We used ‘mccrTest’ to correct gamma statistics for incomplete taxon sampling, the number of replicates was set to 5000. Estimates for the total number of species in the entire Neotropical fraction of the genus *Eois*, as well as in the eight sub-clades with sufficient representation in our data set, were obtained in order to control for incomplete taxon sampling. We used the combined rarefaction-extrapolation algorithm (extrapolating to 500 sequences per clade each) and the Chao1 estimator (both implemented in [54], to come up with conservative estimates of species richness. Correction for estimated total species numbers were performed for the lower, mean and upper estimate respectively (see S3 Table).

### Host plant records and ancestral state estimation

Host plant records for *Eois* used in this study consist of published [11,12,15,55] and previously unpublished data collected by ourselves (see [12] for methods). New host plant data included in this study resulted from quantitative and qualitative surveys in southeastern Ecuador conducted between 2007 and 2013. Due to the poor state of taxonomy of *Eois* moths as well as *Piper* plants we were unable to include records published by other workers as no reliable taxon assignment is possible. Altogether, hostplant records were available for 76 *Eois* BINs included in this study. Reconstruction of ancestral states was performed along with estimation of phylogeny with BEAST v1.8.2. Hostplant data was imported as a discrete trait partition in BEAUTi. See S4 Table for the applied coding scheme. To allow parsing of reconstructed state probabilities the BEAST output was parsed using the R package ‘OutbreakTools’ (The Hackout team, 2015) and custom scripts. Node pies were plotted with ‘ggtree’.

### Ancestral range estimation

Even though the available taxon sampling both within *Eois* as well as outgroup taxa is currently limited we provide a tentative coarse-scaled hypothesis for the biogeographic origin of *Eois*. We divided the range of *Eois* and outgroup taxa into six regions (S1 Fig). Distribution of clades was scored in a presence-absence matrix (S5 Table) according to available literature [56]. We performed biogeographic model selection with the R package ‘BioGeoBEARS’ v0.2.1 [57]. The preferred model was selected according to the AICc criterion as calculated by the BioGeoBEARS example script.

## Results

The sequence alignment of the COI gene had a length of 1536bp and the one for the Ef1**α** gene 1240bp, amounting to a combined dataset of 2776bp. No insertions or deletions were detected, except for COI sequences of the two *Timandra* species in the outgroups being shortened by one codon as reported by [58].

### Phylogenetic relationships

#### Phylogenetic relationships within *Eois*

**Fig 1:**
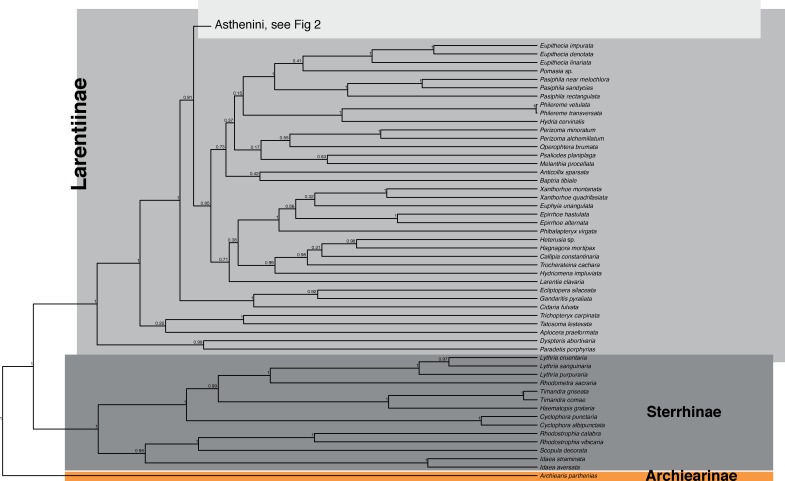
Phylogenetic tree obtained from BEAST. Part 1. Outgroup portion of the phylogenetic tree obtained from BEAST. Bayesian posterior probabilities are given at nodes.

**Fig 2:**
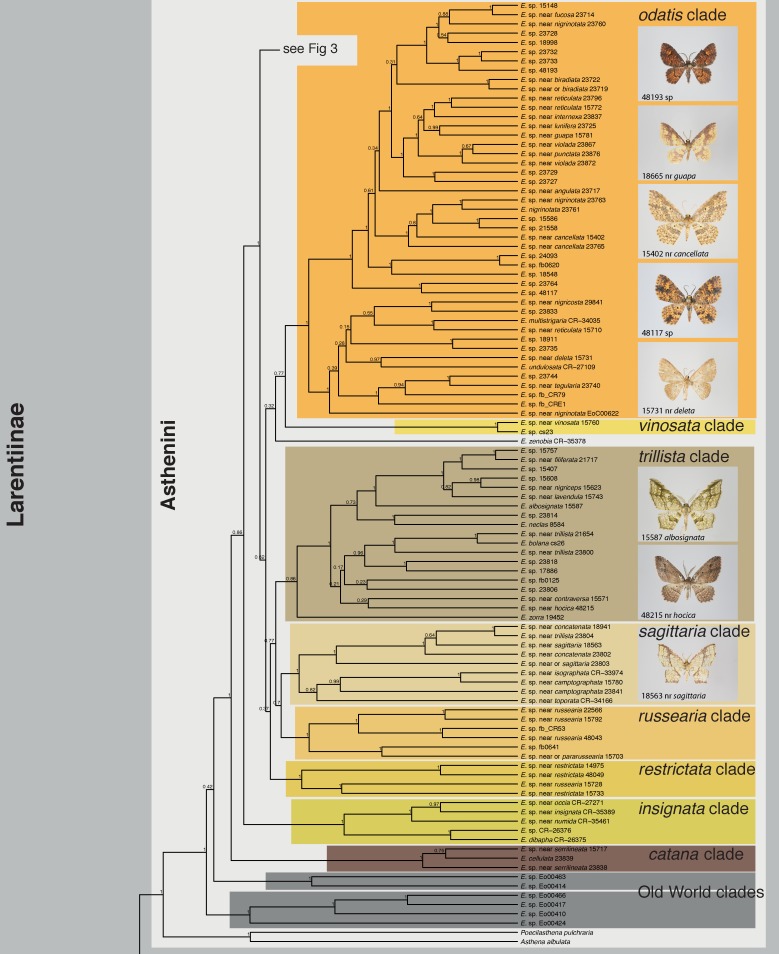
Phylogenetic tree obtained from BEAST, Part 2. All informally named infrageneric clades are indicated along with photographs of select members of each clade within Neotropical *Eois*. Bayesian posterior probabilities are given at nodes.

**Fig 3:**
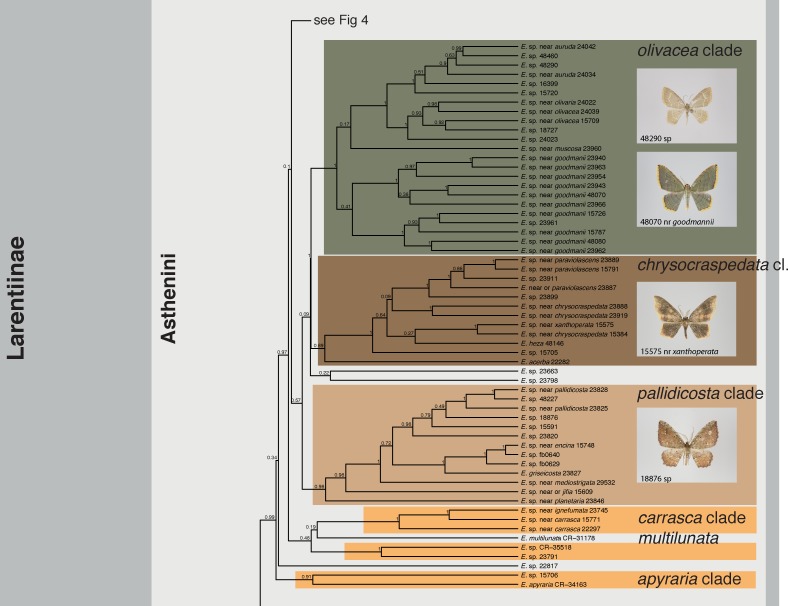
Phylogenetic tree obtained from BEAST, Part 3. All informally named infrageneric clades are indicated along with photographs of select members of each clade within Neotropical *Eois*. Bayesian posterior probabilities are given at nodes.

**Fig 4:**
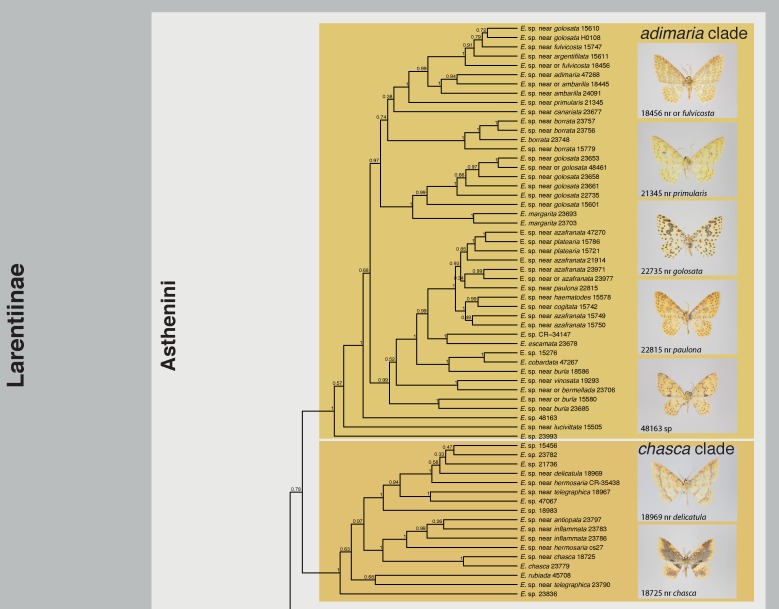
Phylogenetic tree obtained from BEAST, Part 4. All informally named infrageneric clades are indicated along with photographs of select members of each clade within Neotropical *Eois*. Bayesian posterior probabilities are given at nodes.

The maximum clade credibility tree obtained from BEAST is shown in Figs 1–4, the maximum likelihood tree calculated with RAxML in S2 Fig *Eois* was consistently recovered as monophyletic with full or near full support (Bayesian posterior probability (bpp) = 1, maximum likelihood bootstrap (mlb) = 98). The monophyly of Neotropical *Eois* received full to moderate support (bpp = 1; mlb = 72). Monophyly or non-monophyly of Old World *Eois*, in contrast, is highly equivocal. BEAST analyses recovered Old World *Eois* as paraphyletic in respect to Neotropical *Eois*. The ML tree recovered Old World *Eois* as monophyletic albeit with poor support (mlb = 58). The conflicting topology recovered by BEAST where Old World *Eois* are recovered as paraphyletic received only insufficient support (bpp = 0.42). Fifteen well-supported clades of varying size (2–45 species) within the Neotropical *Eois* were recovered and informally named here to facilitate discussion (Figs 1–4, Table 1). Relationships among those clades are in most cases only moderately or even poorly supported. In contrast, internal topology of the clades is in most cases reasonably well supported.

**Table 1:** Overview of clades recognized within Neotropical *Eois* moths. Numbers of species represented in [11] and in the current study are indicated as well as provisional distribution information. A named clade consists of a well-supported monophyletic group comprising at least two species. Shaded in grey: large clades (>10 species). SA: South America, CA: Central America.

clade or species	Strutzenberger et al. 2010	This study	Distribution
**9 clades confirmed**			
*catana* clade	3	3	SA+CA (lowland)
*sagittaria* clade	5	9	SA+CA (montane)
*trillista* clade	4	19	SA, few CA (montane)
*odatis* clade	28	45	SA, few CA (lowland & montane)
*pallidicosta* clade	5	13	SA, few CA (montane)
*chrysocraspedata* clade	7	12	SA, few CA (montane)
*olivacea* clade	16	23	SA, few CA (montane)
*chasca* clade	8	17	SA, few CA (montane)
*adimaria* clade	23	45	SA, few CA (montane)
**3 groupings up-ranked to clade**			
*restrictata* clade	2	4	SA+CA (montane)
*apyraria* clade	1	2	SA+CA (lowland)
*carrasca* clade	2	3	SA, few CA (montane)
**3 newly recognized clades**			
*insignata* clade	-	5	SA+CA (lowland)
*russearia* clade	-	6	SA+CA (lowland)
*vinosata* clade	-	2	SA (montane), any CA?
**individual species, not assigned to named clade**			
*zenobia*	-	1	CA (lowland)
*multilunata*	-	1	SA+CA (montane)
sp. ID 22817	-	1	SA (montane)
sp. ID 23798	-	1	SA (montane)
sp. ID 23663	1	1	SA (montane)
sp. CR-35518	-	1	CA (montane)
sp. ID 23791	-	1	SA (lower montane)
**Total**	**105**	**215**	** **

### Outgroup relationships

The subfamily Larentiinae was firmly recovered as monophyletic (bpp = 1, mlb = 84), equally the Sterrhinae were recovered as monophyletic (bpp = 1; mlb = 70). Both analyses recovered *Eois* as nested within a monophyletic clade with all included representatives of the tribe Asthenini (bpp = 1; mlb = 79).

### Host plant records

Previously unpublished host plant records for 28 species of *Eois* from the Andes of southeastern Ecuador are given in S2 Table. These include 21 new records of species feeding on *Piper*, 5 on *Hedyosmum*, and 2 on *Siparuna* (Siparunaceae). For the core study area in Ecuador these new observations almost double the number of published *Eois* host plant records. This raises the total number of OTUs in this study with available host plant records to 76, with 32% of all host plant records referring to plants other than *Piper*.

### Phylogenetic patterns of host plant use

**Fig 5:**
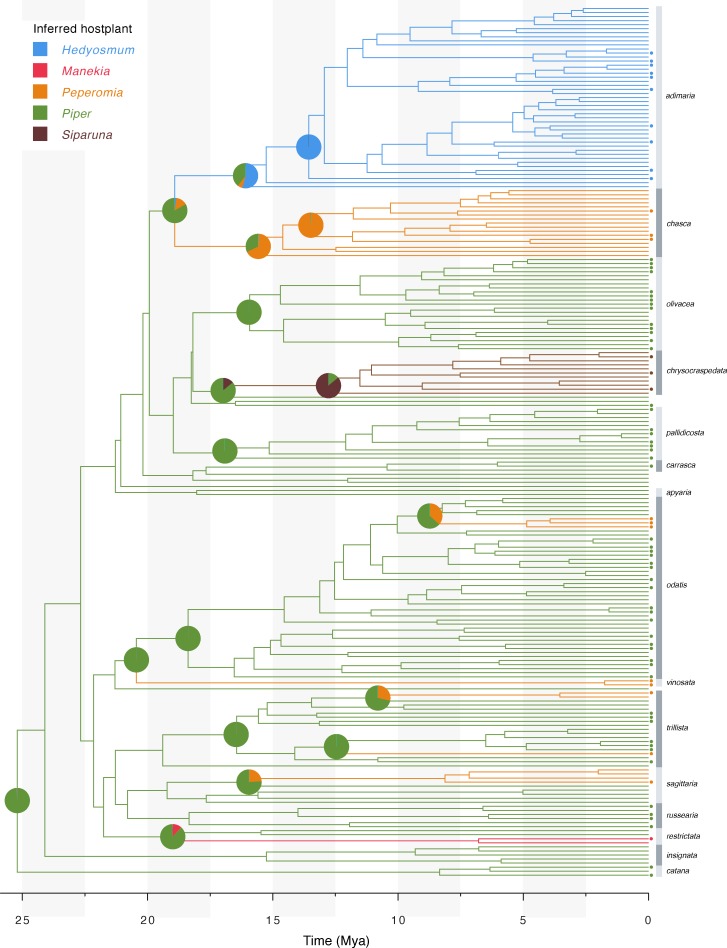
Reconstruction of ancestral host plant use. Branches are colored according to the most likely state, pie charts displaying the probability of all states were plotted at nodes where shifts in host plant use occurred. Dots next to tips indicate the presence of a host plant record for this particular taxon.

Reconstructed ancestral states of host plant use are shown in Fig 5, see S3 Fig for full results of ancestral host plant reconstruction. *Piper* feeding was unambiguously reconstructed as the ancestral host plant association of Neotropical *Eois*. Shifts from *Piper* to other host plants were found to correspond to clade boundaries in the case of the *adimaria* clade (to *Hedyosmum* host plants), the *chasca* and *vinosata* clade (to *Peperomia* host plants), and the *chyrsocraspedata* clade (to *Siparuna* host plants). Five additional shifts from *Piper* to *Peperomia* were inferred to have occurred in the *odatis*, *trillista* and *sagittaria* clades, respectively, making for a total of six independent shifts to feeding on *Peperomia*. One shift to *Manekia* (Piperaceae) was inferred in the *restrictata* clade.

### Molecular dating and analysis of speciation rates

**Fig 6:**
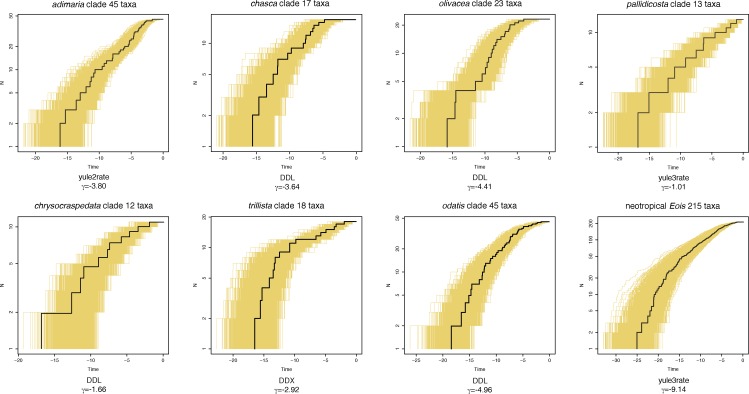
Lineage through time plots. Lineage through time plots for Neotropical *Eois* and for all internal clades containing at least 10 species are presented. The entire BEAST tree sample was plotted for each clade, with the plot resulting from the maximum clade credibility tree superimposed (black). Below each chart the respective best evolutionary model and the value of the pertinent gamma statistics are given.

The split of *Eois* from the remaining Asthenini (i.e. *Asthena*) was estimated to an age of 31.4Ma (26.4–36.3Ma 95%HPD). The age of *Eois* itself was estimated to be 27.5Ma (23.2–32.3Ma 95%HPD). Neotropical *Eois* were dated to an age of 24.2Ma (20.3–28.4Ma 95%HPD). The origin of most clades within Neotropical *Eois* was estimated to lie between 15 and 20 mya. See S4 Fig for the dated tree with all node ages and 95% HPD indicated. Lineage-through-time (LTT) plots for each *Eois* clade containing at least 10 taxa, and for Neotropical *Eois* in total, are given in Fig 6. No correlation in either direction between clade age and species richness could be identified (Spearman’s rank correlation: **ρ** = 0.39, p = 0.15). The best fitting rate model obtained from ‘fitdAICrc’ is indicated for each clade. Five out of seven clades were found to best match a density dependent model. The density dependent logistic model was favored over a density dependent exponential model in all clades but the *lavendula* clade where Akaike weights for both models are too close to favor one over the other. The *adimaria* and *pallidicosta* clade are best described with a yule3rate or yule2rate model, respectively. The entire Neotropical *Eois* also follow a yule3rate model. Shift times of yuleXrate models were all located within the Pleistocene with the exception of the *pallidicosta* clade where a shift to a higher rate occurred in the early Pliocene. See S6 Table for complete fitdAICrc output for each clade. The gamma statistic showed a significant slowdown in diversification in all clades except for the *pallidicosta* and *chrysocraspedata* clades. All significant values retained their statistical significance even when the highest obtained estimates for total species number per clade were used to correct for incomplete taxon sampling with mccrTest.

### Biogeography

The DEC+J model was selected as the best fitting model with an Akaike weight of 0.7 (S7 Table). This model and all other models with the exception of DEC estimated the ancestral range of *Eois* to be Tropical Asia. Alternatively, the DEC model reconstructed a combined ancestral range of Neotropics+Tropical Asia as the most likely ancestral distribution. While the bulk of our data come from the southern Ecuadorian Andes, *Eois* species from Costa Rica represented in our dataset were recovered in the low elevation *insignata* clade towards the base of Neotropical *Eois* as well as interspersed within most other clades or representing one single species lineage (*E*. *zenobia*). See S5 Fig for complete results.

## Discussion

### Phylogenetic analysis

The monophyly of *Eois* as a whole was well supported in both Bayesian and ML trees and so was the monophyly of Neotropical *Eois*. This confirms previous hypotheses based on genital morphology [59] and the results of [11]. We obtained, however, no sufficient support for, or against, the monophyly of Old World *Eois*. The monophyly of this subset appeared well supported in [11] but received virtually no support in the present study. This is likely due to the sample size for Old World *Eois* being identical to the previous study while Neotropical *Eois* and outgroup taxon sampling was twofold increased. An even denser taxon sampling will be required to elucidate the phylogenetic status of Old World *Eois* and determine whether Neotropical *Eois* are nested within, or sister to, Old World *Eois*. The clade consisting of *Asthena* and *Poecilasthena* was recovered as sister group to *Eois* with full support in Bayesian and moderate support in ML analyses. This confirms the assignment of *Eois* to the Asthenini by [43,60] as well as in previous morphology based studies [58,61], whereas [62] had excluded *Eois* from Asthenini on the grounds of characters in genitalia morphology. However, the position of the tribe Asthenini within the subfamily Larentiinae remains unresolved [56]. While [44] identified Asthenini as being sister to a clade comprising Melanthiini, Perizomini and Eupitheciini, [60] found Asthenini being a relatively basal Larentiinae lineage. The monophyly of Asthenini received strong support by [60].

Our results confirm all nine clades of *Eois* defined by [11] as monophyletic entities with similar support values (Figs 1–4, Table 1). Only in the *chrysocraspedata* and in the *trillista* clades, support values decreased slightly because of the inclusion of species that are sister to the remaining members of these clades, respectively. Three previously unnamed lineages could now reliably be defined as clades. In addition, three clades were newly discovered, i.e. none of their members had been represented in any phylogenetic study so far, resulting in a total of 15 informally named clades and five isolated species not assigned to a named clade, but clearly not belonging to any of the other named subgroups. This system of informally named clades facilitates an overview of the genus and will enable a more precise assignment of undescribed species in forthcoming studies; it is also expected to ease species-level taxonomy [9].

### Divergence dating and rates

Most age estimates obtained in the present study turned out to be somewhat younger than previous estimates by [28]. The age of Neotropical *Eois* for example was earlier estimated to be approximately 31Ma while this present study estimated the same clade to be only 24Ma old. This difference is likely caused by denser taxon sampling both within *Eois* and in the outgroups. In spite of these numerical differences the patterns recovered here broadly confirm the earlier findings of [28]. As evident in Fig 6 rapid diversification in all subclades as well as in the entire Neotropical *Eois* clade took place during the central and north-Andean uplift in the late Miocene and Pliocene.

*Eois* clades diversified early and speciation continued at substantial rates until the latest Neogene followed by a pronounced slowdown of net-diversification in the Quaternary. Furthermore, we did not observe any clear differences in radiation patterns between the various clades within Neotropical *Eois*, regardless of their richness, host plant affiliations (see below) or geographical center of distribution. The shapes of LTT plots for all clades with more than 10 species are remarkably similar. This seems to emphasize the influence of large-scale factors like climate change and the Andean uplift compared to micro-habitat and host plant use. The observed slowdown towards the present may either represent a true slowdown of net-diversification or a taxonomic artifact caused by insufficient taxonomic treatment of genuinely young, not yet distinguishable 'cryptic' species. This seems, however, unlikely as the BIN algorithm tends to perform well and if anything is more prone to over-splitting than under-splitting of species [43].As suggested by results obtained from fitting evolutionary rate models a density dependent process may be the cause of this slowdown. A niche filling scenario pertaining to host plant use is certainly possible but currently available data do not allow for further investigation along those lines. On the other hand it is easily imaginable that this slowdown was caused by the dramatic environmental changes that have occurred during the Pleistocene. Instead of facilitating speciation these changes might actually have halted speciation and/or caused increased extinction.

With regard to explaining the mechanisms behind the exceedingly high Andean species richness no definitive statement can yet be made without inclusion of Amazonian lowland *Eois*. A frequently proposed mechanism is the species-pump hypothesis [37], where it is postulated that Andean lineages tend to have higher speciation rates compared to Amazonian lineages. This hypothesis can in most instances be considered synonymous with the ‘cradle’ hypothesis [63,64]. The Andes do indeed provide potential for high speciation rates caused by a high degree of habitat heterogeneity in combination with dispersal barriers created during periods of rapid uplift or climatic oscillations. Present day Andean moth communities are in fact known to substantially differ from another over small geographic and elevational scales [19,20,65]. Small scale vicariance is therefore a very plausible driver of speciation within *Eois*. Furthermore, pulses of even more recent speciation events might be expected due to the climatic oscillations during the Pleistocene, which have triggered vertical movements of biomes. Analyses of pollen deposits have shown that the elevational position of the tree line, and the extent of forest versus grassland vegetation, have substantially varied in the Andes during the past two million years [66]. Later on, during the Pleistocene climatic oscillations, surprisingly few new taxa emerged in this moth group, despite substantial ecological fluctuations that hit the Andes as well as the Amazonian lowlands. The formation and constant re-formation of rivers [31,67], the Pebas and Acre wetland systems [67] and marine incursions [68] provided significant potential for in-situ lowland radiations which may well have contributed to Andean radiations. Both the Pebas and Acre system along with marine incursions occurred concurrently with early cladogenesis in Neotropical *Eois*. Mixed scenarios involving the Andes as species-pump along with repeated faunal exchanges with Amazonia have been identified in butterflies [38] and frogs [69].

*Eois* moths exhibit a clear pattern of Neogene diversification, a pattern found in many Andean taxa, ranging from tetrapod vertebrates [70] to other groups of insects [32,33,71]. Similar scenarios have been identified for ants [64], birds [72], leaf beetles [73] and plants in the order Malpighiales [74]. Results obtained from the gamma statistic are also in accordance with [28]. The observed slowdown is a commonly recovered pattern obtained for other insect taxa as well, e.g. [32].

### Biogeography and ancestral larval hosts

The previous study [11] hinted at an Asian origin of *Eois*. Analyses in this present study provided further support for an Asian origin of *Eois* as the most likely scenario. This is consistent with the predominant geographic distribution of the tribe Asthenini in the Palaearctic, Oriental and Australian regions [62]. Apart from the monospecific genus *Leucoctenorrhoe* there are no Asthenini other than *Eois* known to occur in the Neotropics,. The tribal assignment of *Leucoctenorrhoe* has never been validated in a molecular phylogenetic study, so *Eois* may well comprise the only representatives of the Asthenini in South and Central America. Larval hostplants of the Holarctic-Oriental genera *Asthena*, *Hydrelia* and *Venusia* and the Palaearctic genus *Euchoeca* include a variety of deciduous broad-leaved trees and shrubs (Betulaceae, Fagaceae, Cornaceae, Rosaceae, Ulmaceae, Sapindaceae, Salicaceae, etc.), but none are known to be affiliated with Piperaceae host plants, nor with Euphorbiaceae (the only known host plants of Old World *Eois*). Caterpillars of some Australian representatives of *Epicyme* and *Poecilasthena* feed on plants in the Ericaceae, Haloragaceae, or Myrtaceae [75], and in at least one single instance on Piperaceae [73]. Feeding on Euphorbiaceae is rare in geometrids, apart from Old World *Eois* there are only two known instances of Euphorbiaceae feeders within the Asthenini. *Eschatarchia lineata* was recorded to feed on *Mallotus* (Euphorbiacae) in Japan [76] and *Minoa murinata* is known as a monophagous feeder on *Euphorbia* plants. *Minoa* was placed in the Asthenini by [56] contrary to [62]. These observations indicate that feeding on Euphorbiaceae, while otherwise rare in the Geometridae, repeatedly recurs among close relatives of *Eois*. *Eois* caterpillars (unfortunately not identified to species level) were also regularly found to feed on introduced species of *Piper* (of Neotropical origin) in Papua New Guinea [77], suggesting the presence of some physiological pre-adaptation to utilize this plant family with its characteristic secondary metabolites prior to the colonization of the Neotropics. Inferring whether the shift to Piperaceae at the origin of Neotropical *Eois* occurred from Euphorbiaceae or an unknown (likely woody) hostplant requires more comprehensive data on host plants use in Old World *Eois*.

Our tentative biogeographic hypothesis needs to be tested through denser taxon sampling within Old World *Eois* as our sampling is limited to five Asian and only a single African species. Additional sampling of relevant outgroups such as the Asthenini is also required to refine this initial biogeographic hypothesis. As it stands, the phylogenetic reconstruction is still mostly based on material from a broad elevational range of one region in the Ecuadorian Andes, even though it also contains 15 taxa from Costa Rica. At any rate, not all *Eois* clades are equally represented in different regions of Central and South America. For example, members of the four basal clades (*catana*, *insignata*, *restrictata* and *russearia*) tend to be widely distributed at low elevations in the Neotropical region whereas members of the ‘crown groups’, i.e. the *adimaria*, the *chasca* and the *olivacea* clades, are mostly distributed in montane regions. This stimulates the hypothesis that evolutionary dynamics differed between lowland and highland *Eois*. Addressing this idea will require inclusion of far more taxa from Neotropical lowland sites.

### Host plant associations within *Eois*

From our reconstruction of the history of host plant use it becomes apparent that feeding on Piperaceae is an ancestral character state of Neotropical *Eois* and is also the most widespread larval host affiliation within this species-rich group. However, only two major clades (*pallidicosta* and *olivacea*) were identified to exclusively comprise *Piper* feeders. In other clades (e.g. *odatis* and *trillista*) with widespread associations with *Piper* some species also feed on *Peperomia* [15]. It appears therefore doubtful that *Eois* moths are involved in a large-scale reciprocal co-evolutionary interaction with *Piper* plants. *Eois* is more likely an assemblage of co-evolutionary scenarios limited to smaller clades which may be confined to small geographic scales. The role of spatial and temporal patterns in co-evolution scenarios has been emphasized by [78,79] but remains massively under-investigated in studies on co-evolution. Diversification patterns in all but two sub-radiations within *Eois* were found to best fit a density dependent model. The presence of a slowdown of speciation rates in combination with density-dependence has previously been referred to as one of the hallmarks of adaptive radiations. Either way, our analyses based on a greatly extended data set clearly confirm that in *Eois*, as in many other folivorous insects [80,81], host plant relationships carry strong phylogenetic signal. In particular, at least two host shifts appear to have occurred at the root of two substantial radiations within *Eois*, viz. the striking shift to the comparatively rare and not particularly species rich plant genus *Hedyosmum* [82] in the *adimaria* group, and the complete shift to *Peperomia* (*chasca* group). While our study indicates that host plant shifts from *Piper* to *Manekia*, *Siparuna* and *Hedyosmum* occurred just once, colonizations of *Peperomia* took place at least six times independently. This is probably due to the fact that *Peperomia* is the sister genus of *Piper*. Plant chemistry is in general closely correlated with plant phylogeny [83]. This leads to the assumption of a higher phytochemical similarity between *Piper* and *Peperomia* compared to the other recorded host plant genera. As adaptations to plant chemistry often are a very conservative feature in specialized groups of herbivorous insects [84,85] this would explain why independent host shifts from *Piper* to *Peperomia* have occurred several times. Based on the comprehensive study by [86], host plant records of *Eois* obtained from [15] could be assigned to four *Peperomia* subgenera which belong to two main plant lineages (lineage F with subgenera *Leptorhynchum*, *Micropiper* and *Multipalmata*; lineage D with subgenus *Pseudocupula*). *Eois* species which were observed to feed on more than one *Peperomia* species were recorded on both main lineages. This supports the notion that plant chemistry probably plays a more important role than plant taxonomy [83] in shaping host plant relationships of herbivorous insects. This notion was previously indicated for *Eois* by [16]. However, comprehensive studies on host plant chemistry would be necessary to test this hypothesis.

## Conclusions

This present study has shown that by doubling our taxon sampling we were able to arrive at more specific estimates with regard to the evolutionary history of a highly species rich clade of tropical herbivorous insects, i.e. moths in the genus *Eois*. Yet all major patterns that had been recognized earlier remained largely unchanged. Clades were consistently estimated to be younger, but still many of the radiations occurred in the Neogene and cannot be attributed to Pleistocene climate oscillations. The far higher number of host plant records also showed that these trophic interactions are far more complex than anticipated. In particular, the notion of *Eois* moths generally co-evolving just with *Piper* plants turned out to be overly simplistic. It would be most worthwhile to further extend explorations of phylogeny and evolution of *Eois* moths. One way ahead are studies in additional geographic regions to cover the diversity of species and their interactions more comprehensively. In parallel, it would be interesting to include existing sequence data and host records in a synthesis. In order to achieve that goal the taxonomic affiliation of many of these available records needs to be firmly established. Likewise, elucidating the evolution of host plant use in Old World *Eois* and how exactly the shift to Piperaceae occurred when *Eois* colonized the New World is contingent upon the availability of more comprehensive and detailed data.

## Supporting information

S1 Fig Map of regions used for biogeographical analysis. Region names are as follows: A: North America; B: Neotropics; C: Palaearctic; D: Tropical Africa; E: Tropical Asia; F: Australasia/Oceania.
(PDF)

S2 Fig Best-known likelihood tree obtained from RAxML. Maximum likelihood bootstrap values are annotated at nodes.
(PDF)

S3 Fig Detailed results obtained form ancestral state reconstruction of host plant use. Proportions of all ancestral states are indicated in pie charts for each node.
(PDF)

S4 Fig Detailed results from molecular divergence dating. Median height for each node is given as well as the range of the 95%HPD.
(PDF)

S5 Fig Detailed results obtained from ancestral range analysis with BioGeoBEARS. The best state for each node is indicated. Region codes correspond the S1 Fig and are as follows: A: North America; B: Neotropics; C: Palaearctic; D: Tropical Africa; E: Tropical Asia; F: Australasia/Oceania.
(PDF)

S1 Table PCR and sequencing primers. Primers used for PCR and sequencing are indicated as well as the cycler program used for each primer combination.
(ODS)

S2 Table Sequenced taxa. All taxa included in this study listed with Genbank accession numbers, collection data and host plant records.
(ODS)

S3 Table Detailed results of species number interpolation. Results of species number interpolation are given for Neotropical *Eois* as well as each major clade within *Eois*. Results obtained from mccrTest to test the robustness of gamma values are indicated for each employed method of interpolation.
(ODS)

S4 Table Input data matrix for reconstruction of ancestral host plant use. (ODS)

S5 Table Input matrix for ancestral range analysis with BioGeoBEARS. (ODS)

S6 Table Results of model fitting with fitdAICrc. Only the best fitting model for each clade is shown.
(ODS)

S7 Table Results of model comparison of results obtained from BioGeoBEARS. (ODS)
