# Supplementary figures and images for: Diversification rates, host plant shifts and an updated molecular phylogeny of Andean Eois moths (Lepidoptera: Geometridae)
Source: PLoS One. 2017 Dec 27;12(12):e0188430. doi: 10.1371/journal.pone.0188430 (PMC5744940; doi:10.1371/journal.pone.0188430)

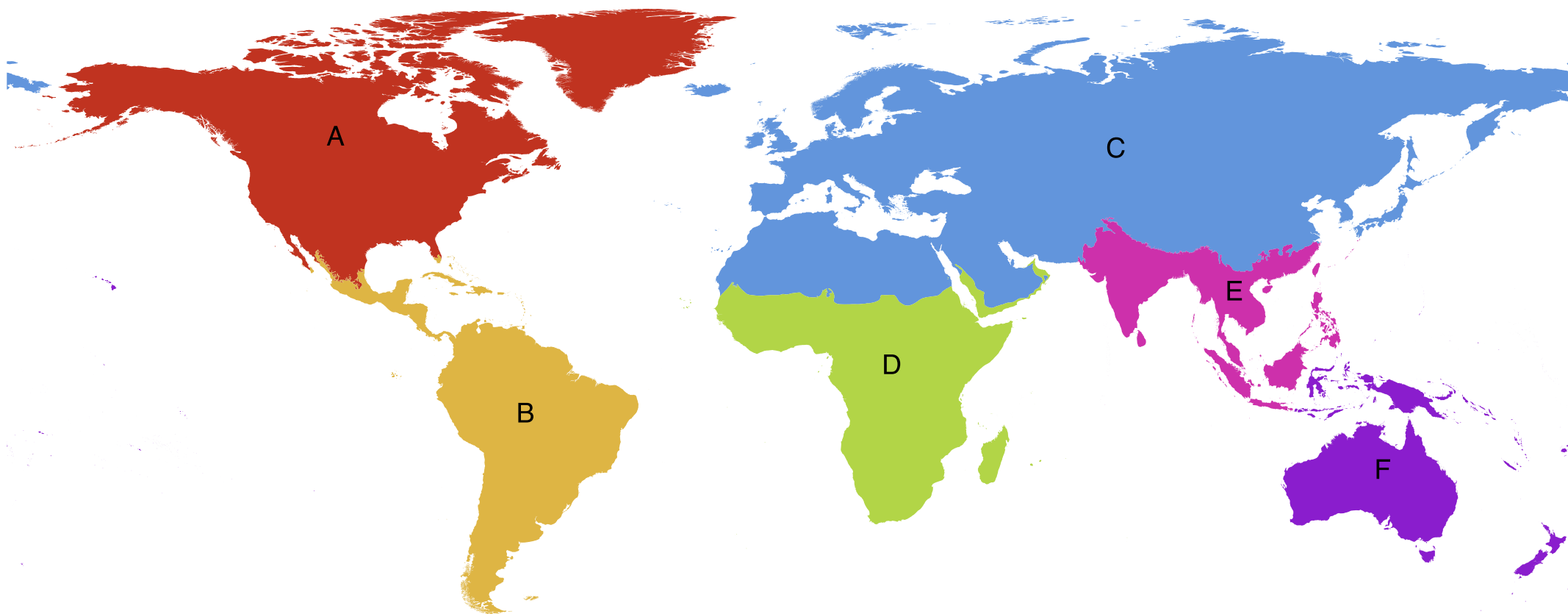

Supplement: S1 Fig — Region names are as follows: A: North America; B: Neotropics; C: Palaearctic; D: Tropical Africa; E: Tropical Asia; F: Australasia/Oceania. (PDF) [file pone.0188430.s001.pdf]

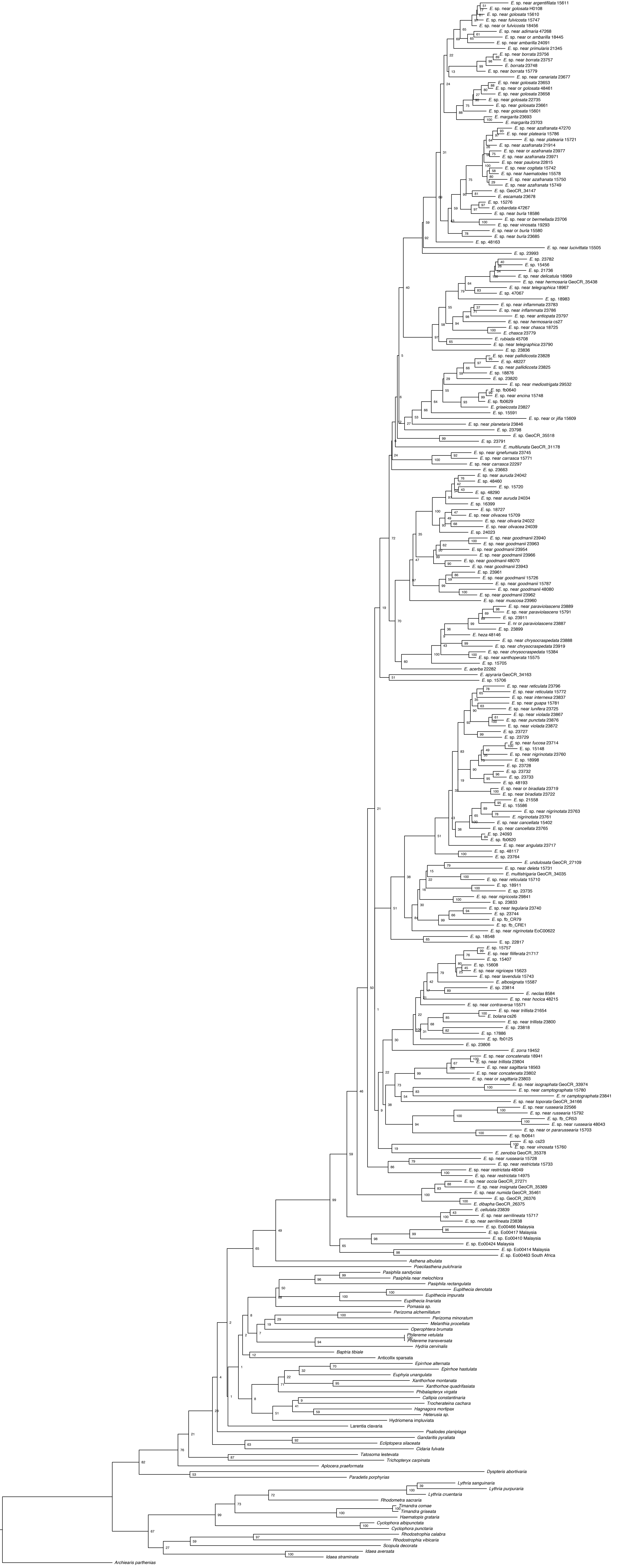

Supplement: S2 Fig — Maximum likelihood bootstrap values are annotated at nodes. (PDF) [file pone.0188430.s002.pdf]

Host plant

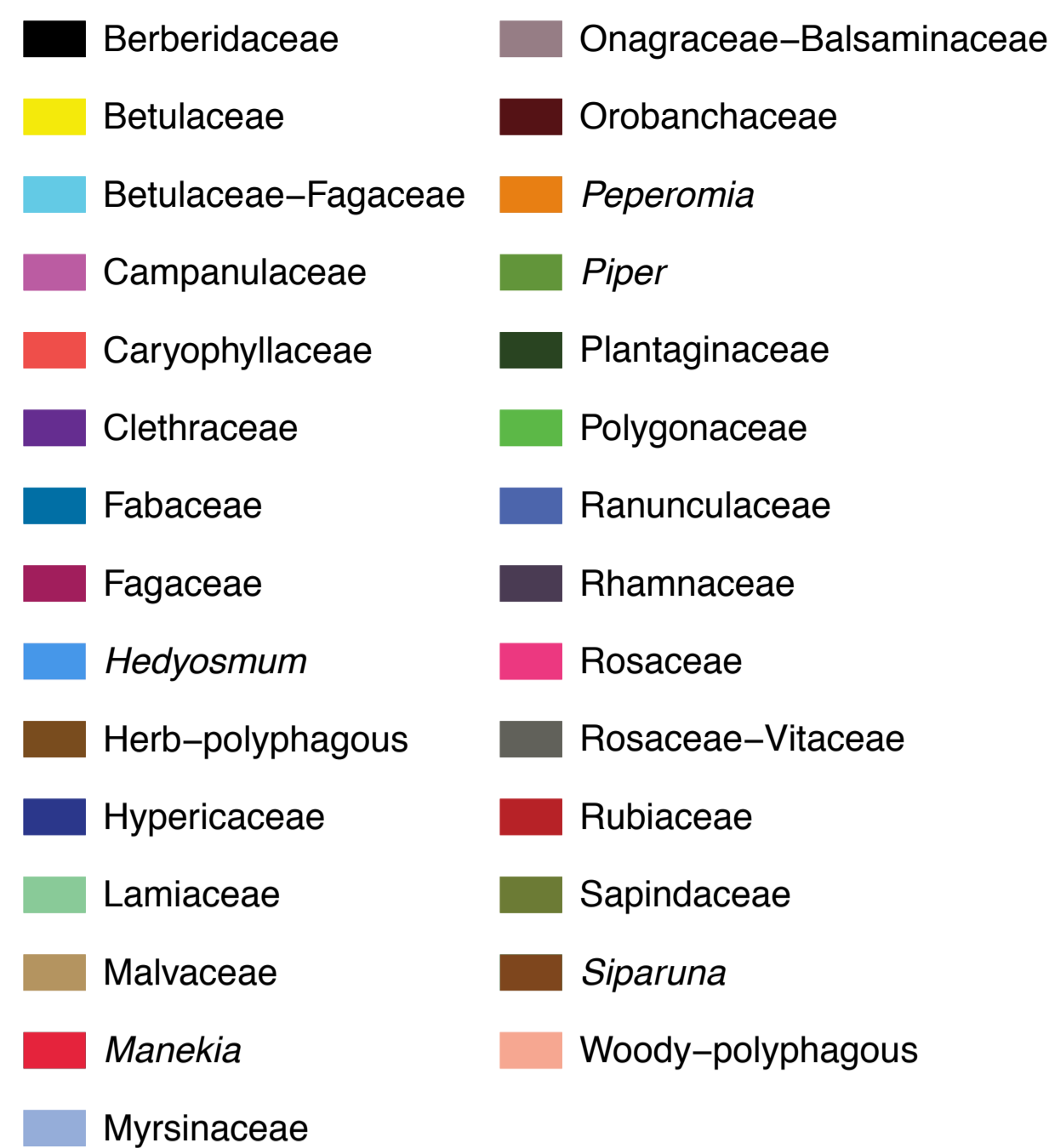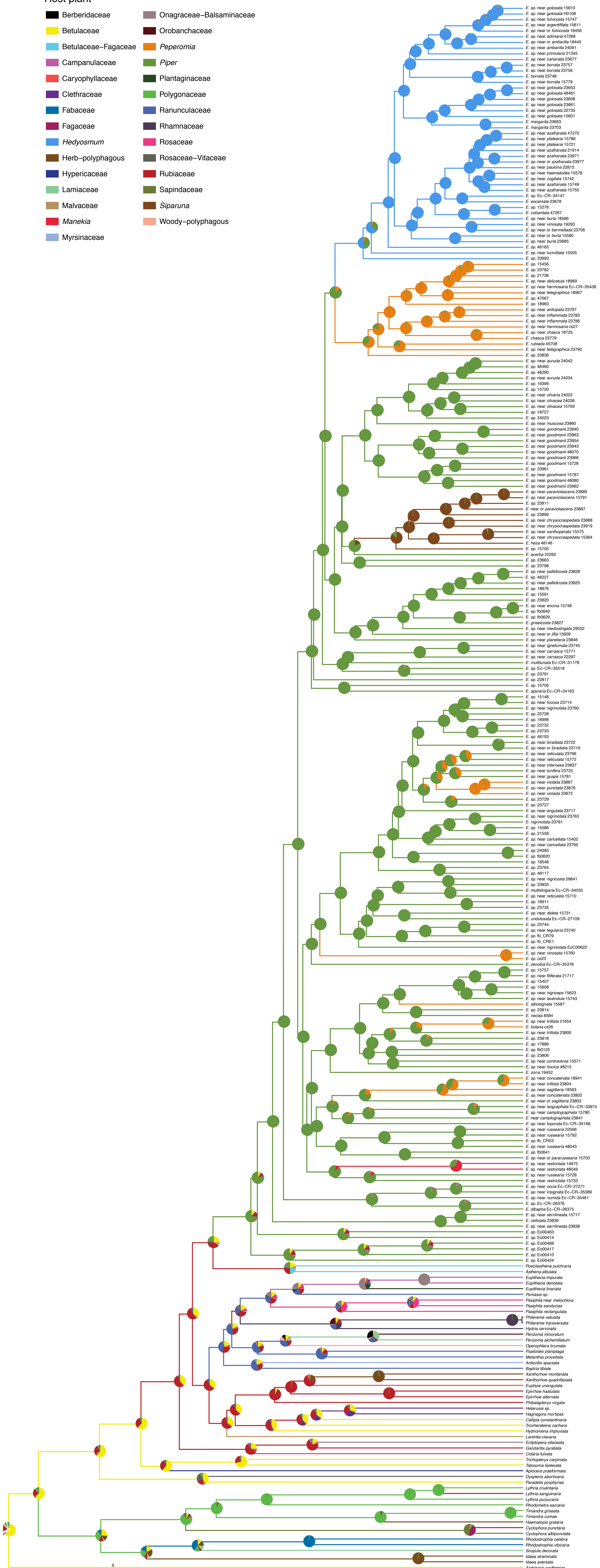

Supplement: S3 Fig — Proportions of all ancestral states are indicated in pie charts for each node. (PDF) [file pone.0188430.s003.pdf]

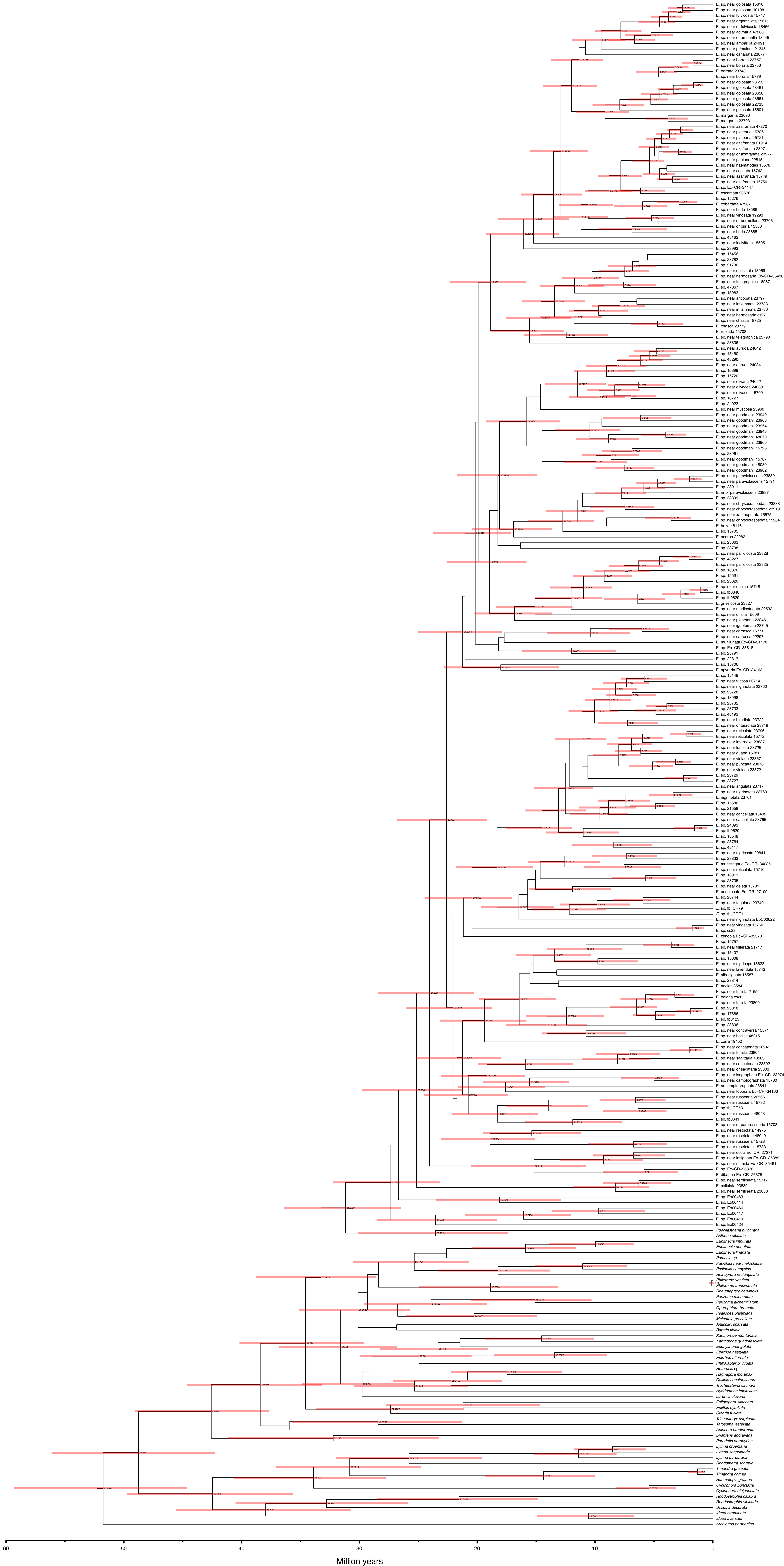

60 50 40 30 20 10 0  
Million years

Supplement: S4 Fig — Median height for each node is given as well as the range of the 95%HPD. (PDF) [file pone.0188430.s004.pdf]

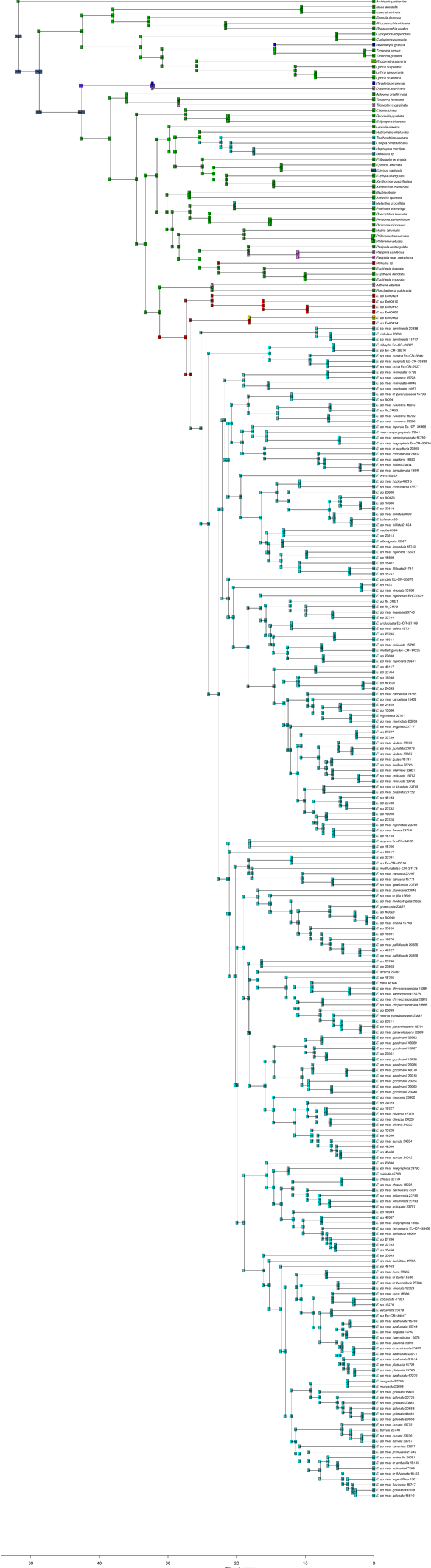

Supplement: S5 Fig — The best state for each node is indicated. Region codes correspond the S1 Fig and are as follows: A: North America; B: Neotropics; C: Palaearctic; D: Tropical Africa; E: Tropical Asia; F: Australasia/Oceania. (PDF) [file pone.0188430.s005.pdf]
